# Microbial production of rhamnolipids using sugars as carbon sources

**DOI:** 10.1186/s12934-018-0938-3

**Published:** 2018-06-08

**Authors:** Yun Nian Tan, Qingxin Li

**Affiliations:** 1 School of Chemical and Biomedical Engineering, Nanyang Technological University, 62 Nanyang Drive, Singapore, 637459 Singapore; 2 Institute of Chemical and Engineering Sciences, Agency for Science, Technology and Research, 1 Pesek Road, Singapore, Jurong Island 627833 Singapore

**Keywords:** Rhamnolipids, Cellulose, Waste, Biotransformation, Fermentation

## Abstract

Rhamnolipids are a class of biosurfactants with effective surface-active properties. The high cost of microbial production of rhamnolipids largely affects their commercial applications. To reduce the production post, research has been carried out in screening more powerful strains, engineering microbes with higher biosurfactant yields and exploring cheaper substrates to reduce the production cost. Extensive refining is required for biosurfactant production using oils and oil-containing wastes, necessitating the use of complex and expensive biosurfactant recovery methods such as extraction with solvents or acid precipitation. As raw materials normally can account for 10–30% of the overall production cost, sugars have been proven to be an alternative carbon source for microbial production of rhamnolipids due to its lower costs and straightforward processing techniques. Studies have thus been focused on using tropical agroindustrial crop residues as renewable substrates. Herein, we reviewed studies that are using sugar-containing substrates as carbon sources for producing rhamnolipids. We speculate that sugars derived from agricultural wastes rich in cellulose and sugar-containing wastes are potential carbon sources in fermentation while challenges still remain in large scales.

## Introduction

Microorganisms such as yeast, bacteria or fungi can produce biosurfactants-surface-active compounds using different substrates such as oils, glycerol, alkanes, sugars and wastes [1–3]. Biosurfactants are biodegradable, making them an attractive alternative to chemically synthesized surfactants which are normally petroleum-based and environmentally hazardous [2, 4–8]. Biosurfactants produced by microorganisms are classified into five major groups including glycolipids, lipopolysaccharides, lipopeptides, phospholipids and fatty acids [9].

Rhamnolipids, a class of glycolipid biosurfactants, are composed of one or two l-rhamnose molecules linked with one or two β-hydroxy fatty acids [10–12]. They are used in various fields such as hydrocarbon degradation, microbial enhanced oil recovery, metal remediation, plant pathogen elimination, bio-pesticides, wound healing and skin treatment therapeutics [2, 8, 13]. Rhamnolipids are predominantly produced by *Pseudomonas aeruginosa* (*P. aeruginosa*) while other bacteria such as *P. chlororaphis*, *P. plantarii*, *P. putida*, *P. fluorescens* and *Burkholderia thailandensis* E264 also produce rhamnolipids [14–16]. In addition to the screened strains from different environment, fungus and engineered bacteria are able to produce rhamnolipids [17]. Rhamnolipids producing related enzymes can be introduced to bacteria and yeast to create strains which use sugars and other carbon sources for rhamnolipid production [18–25].

High production costs pose as the major obstacle to the widespread usage of biosurfactants [26]. Strategies to make biosurfactants commercially competitive include accessing agro-industrial wastes as cheap feedstock, developing overproducing robust wild-type or engineered strains, optimizing fermentation and downstream processes and combining production of biosurfactants with other biomolecules such as enzymes or bioplastics [12, 27]. Here we review the latest progress in microbial production of rhamnolipids using sugars derived from industrial wastes as the carbon sources.

## From sugar to rhamnolipids

**Fig. 1:**
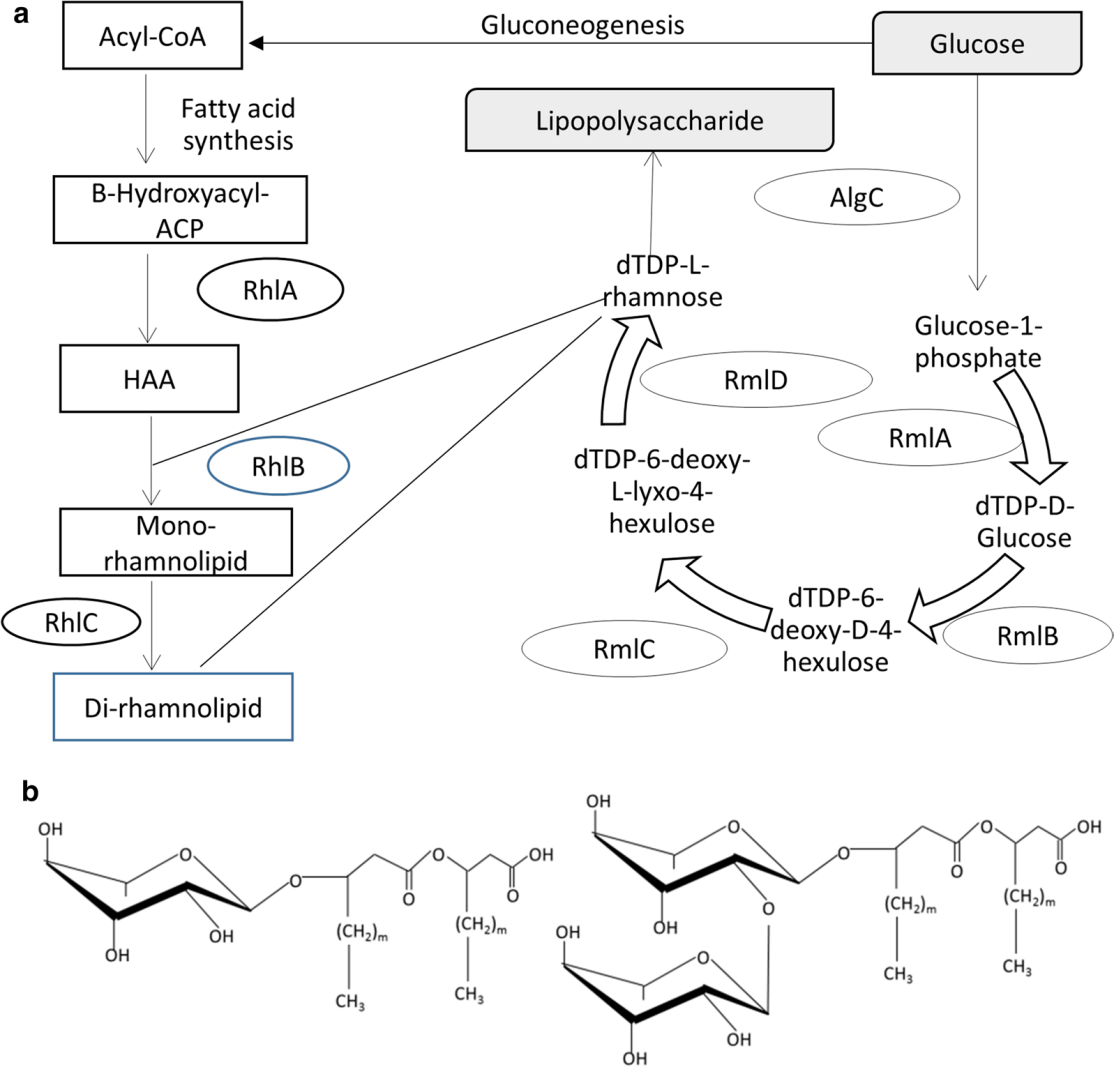
Rhamnolipids synthesis from glucose. **a** Glucose can be converted into dTDP-l-rhamnose which serves as the sugar moiety and Acyl-CoA which can be produced from glucose and converted into the hydrophobic moiety. Some important enzymes such as glucose-1-phosphate thymidyltransferase (RmlA), dTDP-d-glucose-4,6-dehydratase (RmlB), dTDP-4-keto-6-deoxy-d-glucose-3,5-epimerase (RmlC), and dTDP-4-keto-l-rhamnose reductase (RmlD) [149] are shown. **b** Chemical structures of mono- and di-rhamnolipid

The metabolic pathway of rhamnolipids production in *Pseudomonas* has been well characterized [28–31]. Glucose can be used for microbial production of rhamnolipids (Fig. 1) as it can be converted into the precursors required for rhamnolipids synthesis. Glucose can be converted into the sugar moiety—deoxythymidine diphosphate (dTDP)-l-rhamnose. The enzymes required for catalysis are present in most bacteria [32]. For the hydrophobic moiety, the precursor—Acetyl coenzyme A (Acyl-CoA) for fatty synthesis can be obtained from glucose [28]. In addition, the unique rhamnosyltransferase RhlA in *Pseudomonas* is required for the synthesis of the hydrophobic moieties such as 3-(3-hydroxyalkanoyloxy) alkanoic acid (HAA) [22, 33]. To obtain mono- and di-rhamnolipids, RhlB and RhlC are indispensable for the catalysis [30, 34] (Fig. 1). In addition to glucose, other sugars such as lactose can serve as carbon sources for biosurfactant production. It has been noted that rhamnolipids synthesis is a complicated process and regulated by other pathways [35]. Supplying glucose in the medium can not guarantee rhamnolipids production. Fermentation control is still required as rhamnolipids production in an inducible process. Bacterial quorum sensing (QS) system is one of the regulators that affect rhamnolipids production, which requires signal molecules and modulators [12, 29, 35–37]. The QS system is a complicate system and regulators that are affecting rhamnolipids production have been reviewed in several literatures [38–40].

## Microbial production of rhamnolipids using sugar-containing substrates

**Fig. 2:**
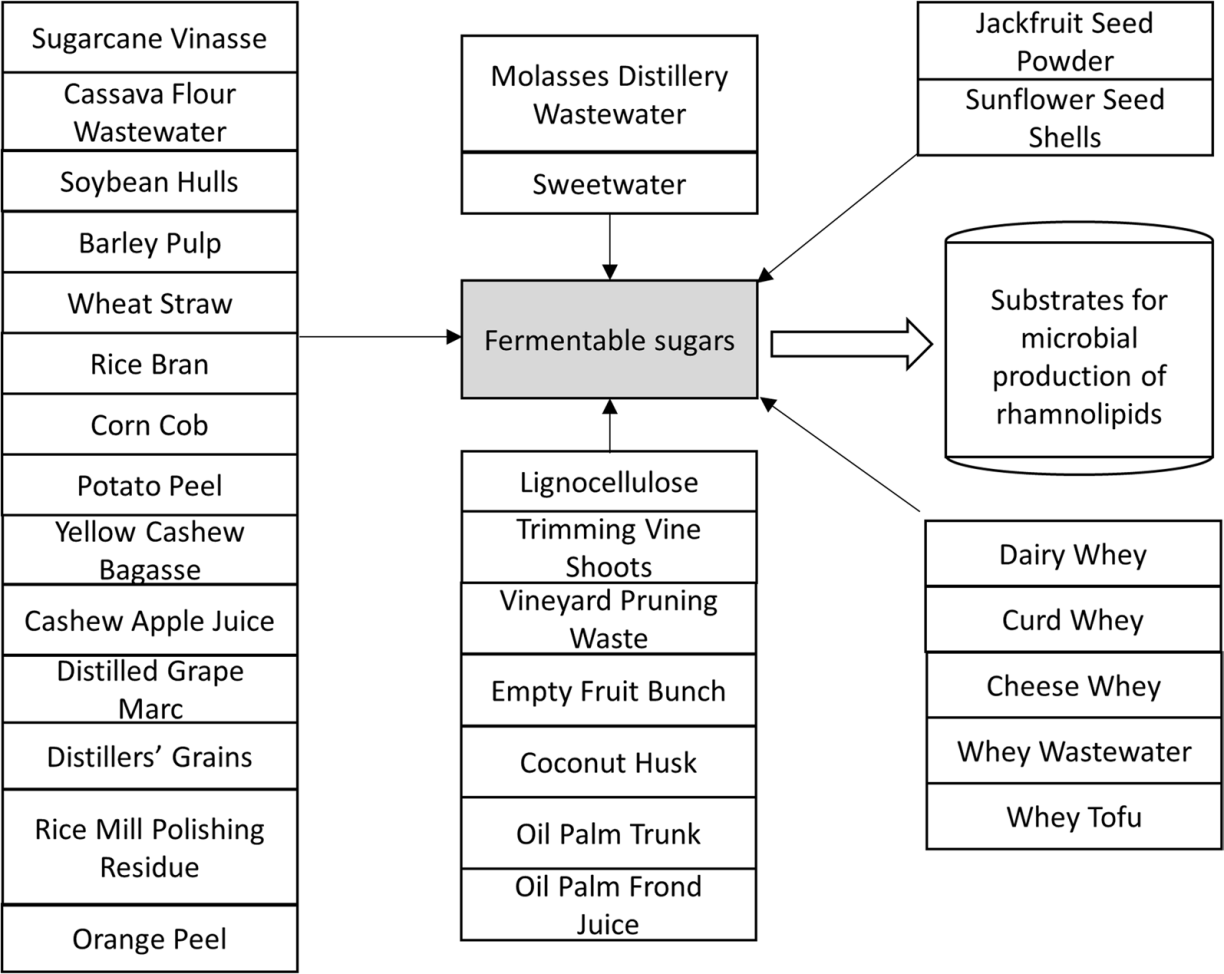
Sugar-containing wastes that can be used as carbon sources for rhamnolipids production. Many wastes contain high amounts of sugars or can be converted into sugars using chemical and enzymatic reactions

Carbon sources including sugars, glycerol, n-alkanes, oils and polycyclic aromatic hydrocarbons have been used for microbial production of rhamnolipids with various yields [41–45]. Currently, the highest rhamnolipids yield is obtained from oil-type carbon sources as they can be easily degraded through the β-oxidation pathway [46]. Recently, interest has arisen in using sugar-containing media as potential substrates for rhamnolipids production despite the lower yields [12]. The cost of sugar-containing wastes is lower than that of oil- or glycerol-containing wastes [47]. In addition, rhamnolipids are strong emulsifiers and extensive organic solvent extraction is required for product separation and purification from oily substrates [36, 48]. Sugar-containing substrates are shown to serve as a carbon source for rhamnolipids production (Fig. 2, Table 1). The components of these substrate are complicated while sugars are the major component (Table 1). It has been noted that other residue components such as proteins, amino acids or lipids might be important for the rhamnolipids production (Table 1).

**Table 1:** Biosurfactant production using sugar-containing wastes

Sugar source	Strain	Biosurfactant yield	References
Barley pulp	*P. aeruginosa* ATCC 9027	9.3 g/L	[73]
	*P. pachastrellae* LOS20	9.2 g/L	[73]
	*P. putida* IBS036	2.4 g/L	[73]
Barley bran husk	*Lactobacillus pentosus*	0.28 g/g biomass	[94]
Bean cake	*Bacillus amyloliquefaciens* XZ-173	2.18 mg/gds	[139]
	*Bacillus amyloliquefaciens* XZ-173	14.61 mg/gds	[140]
Cashew apple juice	*Acinetobacter calcoaceticus*	N.A.	[75]
Yellow cashew	*P. aeruginosa*	7.1–9.3 g/L	[74]
Cassava wastewater	*P. aeruginosa*	169.9–300.3 mg/L	[104]
	*P. fluorescens*	N.A.	[103]
	*Pseudozyma tsukubaensis*	8.11 g/L	[105]
Corncob hydrolysate	*Starmerella bombicola*	33.7–49.2 g/L	[141]
	*B. subtilis* BS-37	523 mg/L	[142]
	*Lactobacillus pentosus*	0.53 g/g biomass	[94]
Corn flour	*Bacillus amyloliquefaciens* XZ-173	1.56 mg/gds	[139]
	*Bacillus amyloliquefaciens* XZ-173	8.38 mg/gds	[140]
Eucalyptus globulus chips	*Lactobacillus pentosus*	0.54 g/g biomass	[94]
Raw cheese whey	*P. aeruginosa* ATCC 10145	9.6 g/L	[99]
Paneer whey	*P. aeruginosa* SR17	2.7–4.8 g/L	[98]
Whey wastewater	*Yarrowia lipolytica* MFW5	N.A.	[101]
	*Micrococcus luteus* MFW1	N.A.	[101]
	*Burkholderia cepacia* MFW2	N.A.	[101]
Curd whey	*P. aeruginosa* BS2	0.92 g/L	[51]
	*P. aeruginosa* BS-P	1.63 g/L	[78]
Distillers’ grains	*Bacillus amyloliquefaciens* MT45	1.04 g/L	[143]
	*Bacillus amyloliquefaciens* MT45 & X82	3.4 g/L	[143]
Distilled grape marc hydrolysate	*Lactobacillus pentosus*	N.A.	[80]
Distillery waste	*P. aeruginosa* BS2	0.91 g/L	[51]
	*P. aeruginosa* BS-P	1.42 g/L	[78]
Hazelnut pulp	*P. aeruginosa* ATCC 9027	11.1 g/L	[73]
	*P. pachastrellae* LOS20	5.4 g/L	[73]
	*P. putida* IBS036	8.5 g/L	[73]
Molasses	*P. aeruginosa* GS3	0.24 g/L	[55]
	Marine *P. aeruginosa*	3.4–3.9 g/L	[63]
	*Bacillus licheniformis* TR7	3.3 g/L	[56]
	*B. subtilis* SA9	3.78 g/L	[56]
	*P. fluorescens*	N.A.	[53]
	*B. subtilis* R1	N.A.	[144]
	*Bacillus licheniformis* K51	N.A.	[144]
	*B. subtilis* 20B	N.A.	[144]
	*Bacillus* HS3	N.A.	[144]
	*B. subtilis* MTCC 1427	N.A.	[54]
	*B. subtilis* MTCC 2423	N.A.	[54]
Date molasses	*B. subtilis* B30	0.3 g/L	[145]
Molasses distillery wastewater	*P. aeruginosa* GIM32	2.6 g/L	[50]
Orange peel	*P. aeruginosa* MTCC 2297	9.18 g/L	[77]
Potato peel	DGEF01-06	N.A.	[76]
Rapeseed meal	*Bacillus amyloliquefaciens* XZ-173	2.68 mg/gds	[139]
	*Bacillus amyloliquefaciens* XZ-173	15.16 mg/gds	[140]
Rice mill processing residue	*B. subtilis* MTCC 2423	4.17 g/kg substrate	[146]
Soy pulp	*Bacillus pumilus* UFPEDA 448	809 mg/L	[147]
Soybean flour	*Bacillus amyloliquefaciens* XZ-173	4.39 mg/gds	[139]
	*Bacillus amyloliquefaciens* XZ-173	38.42 mg/gds	[140]
Soy molasses	*P. aeruginosa* ATCC 10145	11.7 g/L	[49]
Whey tofu	*P. fluorescens*	N.A.	[102]
Sugar beet molasses	*P. luteola* B17	0.53 g/L	[59]
	*P. putida* B12	0.52 g/L	[59]
Sugarcane bagasse	*P. aeruginosa* ATCC 10145	9.1 g/L	[57]
Sugarcane vinasse	*P. aeruginosa* PA1	2.7 g/L	[58]
Sunflower pulp	*P. aeruginosa* ATCC 9027	5.3 g/L	[73]
	*P. pachastrellae* LOS20	5 g/L	[73]
	*P. putida* IBS036	6.7 g/L	[73]
Sunflower seed shell	*Pleurotus djamor*	10.2 g/L	[148]
Sweetwater	Marine *P. aeruginosa*	4.0–4.7 mg/L	[63]
Trimming vine shoots	*Lactobacillus pentosus*	0.71 g/g biomass	[94]
	*Lactobacillus pentosus*	N.A.	[95]
Vineyard pruning waste	*Lactobacillus paracasei*	N.A.	[79]
Wheat bran	*Bacillus amyloliquefaciens* XZ-173	2.74 mg/gds	[139]
	*Bacillus amyloliquefaciens* XZ-173	13.17 mg/gds	[140]
Wheat straw	*P. aeruginosa* NCIM 2036	9.38 g/L	[6]

### Molasses

Molasses is a byproduct rich in sugars. Soy molasses are generated during soybean processing and composed of carbohydrates, minerals, fats, lipids and others. Soy molasses contain mixture of sugars and have been used for rhamnolipids production using *P. aeruginosa* ATCC 10145, giving a rhamnose concentration of 6.9 g/L and biosurfactant concentration of 11.7 g/L [49]. Molasses distillery wastewater was used as a substrate for rhamnolipids production by *P. aeruginosa* GIM32 and the yield reached 2.6 g/L [50]. *Pseudomonas aeruginosa* strain BS2 was able to use distillery waste from the alcohol industry and curd whey waste from the milk industry as substrates for rhamnolipids production and the yields reached 0.91 and 0.92 g/L, respectively [51].

Sugarcane molasses is the final effluent of sugar refinement and comprises approximately 40% (w/w) sugars. The molasses is normally used as an ingredient in some food products and it has impact on immune system [52]. Adding molasses into the medium is able to increase rhamnolipids production by *P. fluorescens* and enhance phenol degradation [53]. Other bacteria such as *Bacillus subtilis* (*B. subtilis*) (MTCC 2423 and MTCC1427) were reported to utilize sugarcane molasses for biosurfactant production at 45 °C [54]. The yield of rhamnolipids reached 0.24 g/L when *P. aeruginosa* GS3 was grown in a medium that contained molasses and corn steep liquor [55]. *Bacillus licheniformis* TR7 and *B. subtilis* SA9 grown on molasses produced biosurfactant at 3.3 and 3.78 g/L, respectively [56]. In addition, exploded sugarcane bagasse has been utilized to co-produce rhamnolipids (9.1 g/L) and ethanol (8.4 g/L) using *Saccharomyces cerevisiae*, *P. aeruginosa* and crude enzyme complexes (CECs) [57]. The CECs were produced by *Aspergillus niger* in solid-state fermentation using different levels of exploded sugarcane bagasse, rice bran and corn cob as substrates [57].

### Sugarcane vinasse

Sugarcane vinasse, a residue from bioethanol production is a common waste during fermentation using substrate from sugar crops. It also contains sugars and is able to serve as a substrate for rhamnolipids production [58]. *Pseudomonas aeruginosa* PA1 could produce 2.7 g/L of rhamnolipids when sugarcane vinasse was used as a substrate in submerged fermentation [58]. *Pseudomonas luteola* B17 and *P. putida* B12 grown on autoclaved medium consisting of sugarcane beet molasses mixed with distilled water, gave maximum rhamnolipids production at 72 h [59]. Other strains such as *B. subtilis* can also use sugarcane vinasse as sources for biosurfactant and energy production [60–62]. In addition to vinasse and molasses, sugar cane refining by-products such as sweet water have also been used as carbon sources for *P. aeruginosa* with rhamnolipids yields of 4.0–4.7 g/L [63].

### Lignocellulose

**Fig. 3:**
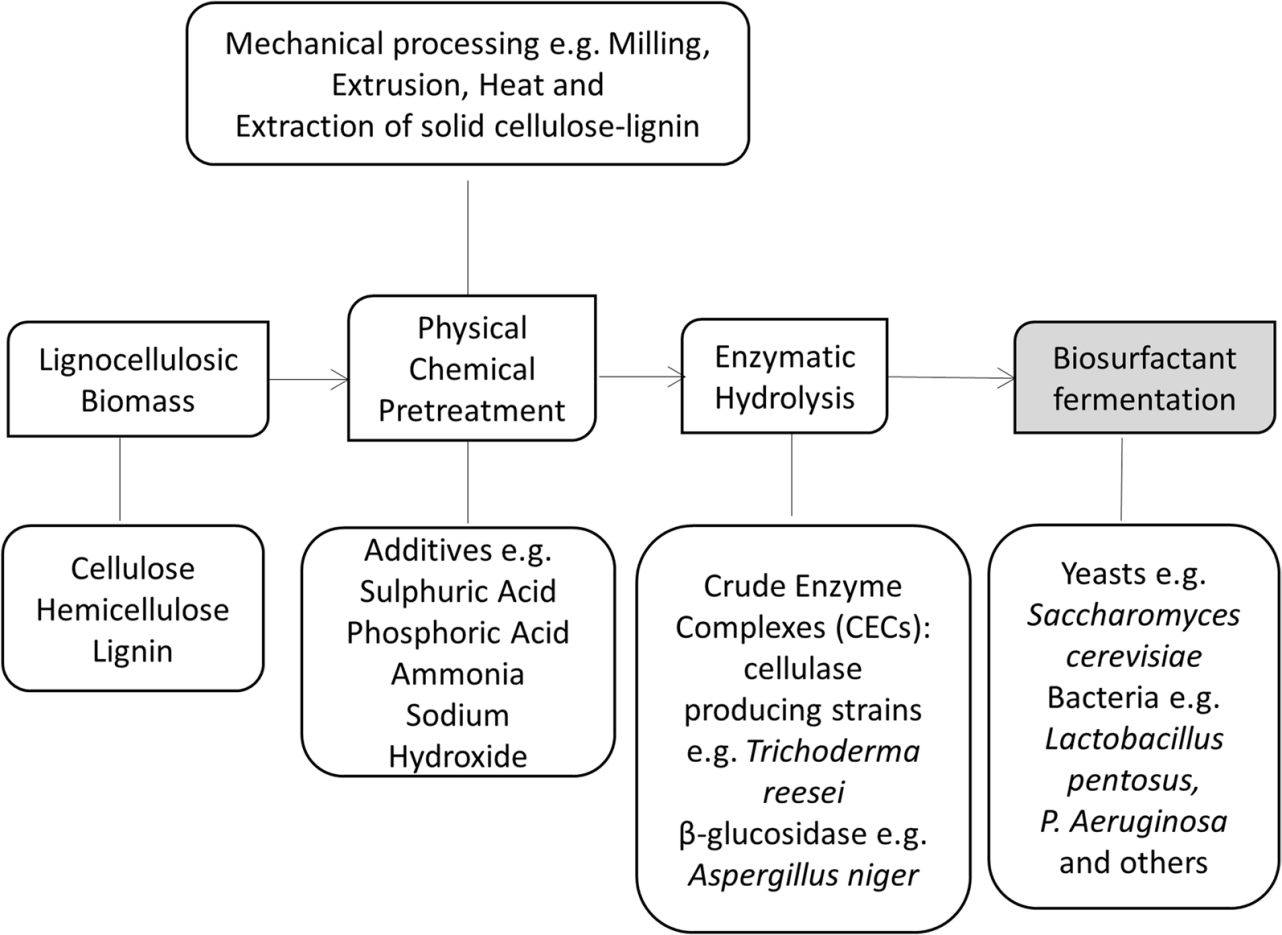
Treatment of cellulose to obtain fermentable sugars for biosurfactant production. Pretreatment of cellulose is required to obtain fermentable sugars. Normally both acid pretreatment and enzymatic hydrolysis are required for sugar production. The produced sugars can be used for microbial production of rhamnolipids

Lignocellulose is present in agricultural products, which makes many related wastes or byproducts attractive low-cost substrates for biosurfactant production. Lignocellulosic biomass has been used as an alternative cost-effective substrate for the microbial production of rhamnolipids as it can be converted into fermentable sugars. It has been noted that pretreatment is required to convert lignocellulosic biomass to fermentable sugars because several enzymes are required for the cellulose degradation (Fig. 3). Converting cellulose to fermentable sugars has been well studied. Pretreatment-a step to obtain cellulose is required, which is a critical step for later enzymatic process as the lignin and other components may affect enzymatic activities or prevent enzyme from accessing cellulose [64, 65]. Cell-degrading enzymes or stains will be mixed with cellulose to obtain fermentable sugars which can serve as carbon sources for microorganisms [66]. While lignocellulosic biomass pretreatment is an important topic, it has been introduced in literatures [67–72] and will not be elucidated here.

#### Wheat straw

Pretreatment of wheat straw with sulphuric acid, phosphoric acid and ammonia and then followed by enzymatic hydrolysis with cellulases from *Trichoderma reesei* NCIM 1186 could obtain sugars [6]. The resulting sugars were used to produce rhamnolipids (9.38 g/L) by *P. aeruginosa* NCIIM 2036 [6]. Similar to wheat straw, lignocellulose-containing wastes such as barley pulp have been used for rhamnolipids production using *P. aeruginosa* ATCC 9027 and the yield of rhamnolipids reached 2.4 g/L [73]. Addition of glycerol to the media could increase rhamnolipids yield to 9.3 g/L [73].

#### Waste from fruit products

In addition to wheat straw, yellow cashew fruit bagasse was crushed into powder and mixed with basal mineral medium for rhamnolipids production using *P. aeruginosa* [74]. Cashew apple juice was shown to serve as a medium for *Acinetobacter calcoaceticus* growth and biosurfactant production [75]. Potato peel mixed with urea was able to affect biosurfactant production positively [76]. *Pseudomonas aeruginosa* ATCC 2297 could produce 9.18 g/L of rhamnolipids using orange peel as carbon sources [77]. Fruit-processing waste and sugar industry effluent were also found to be viable substrates for biosurfactant fermentation by *Kocuria turfanesis* and *P. aeruginosa* [78]. As part of whole waste recycling, vineyard pruning waste was collected and acid hydrolysis was performed to remove the hemicellulosic sugars [79]. The remaining lignocellulosic fraction was undergoing delignification. The cellulosic fraction was then subjected to enzymatic hydrolysis using cellulase and β-glucosidase to obtain cellulosic sugars. The sugars were then used in fermentation as a low-cost carbon source for biosurfactant production by *Lactobacillus paracasei* [79]. Distilled grape marc was also discovered to be a low-cost feedstock of sugars for biosurfactant production by *Lactobacillus pentosus*, with values of relative emulsion volume close to 50% and stabilizing capacity values to maintain the emulsion at 99% [80].

#### Byproducts and wastes from oil production

Oil palm trees are currently very abundant in Southeast Asia for palm oil production and the wastes generated are an untapped resource for biosurfactants production. The byproducts from oil production are rich in cellulose. For example empty fruit bunch (EFB) was converted to fermentable sugars using dilute sulphuric to solubilize hemicellulose and cellulase enzymes produced by *Trichoderma reesei* RUT-C30 (ATCC 56765) to hydrolyze cellulose giving a total sugar yield of 82% [65]. The product was proven to be suitable for cell growth and serve as a potential medium for rhamnolipids production [65]. Palm kernel cake, a solid residue that remains after oil extraction from the kernels of palm fruits, has been identified as a potential low-cost substrate for biosurfactant production due to its high mineral, protein and fatty acid contents [81]. *Providencia alcalifaciens* SM03 was isolated from degraded palm kernel cake and shown to produce glycolipids at 8.3 g/L [81]. A study made use of 25% (v/v) palm oil decanter cake mixed with 1% monosodium glutamate as an optimized media in microbial cultivation [82]. *Ochrobactrum anthropi* 2/3 was able to produce 4.52 g/L glycolipid biosurfactant after cultivation at 30 °C for 96 h [82].

Palm oil mill effluent is a waste by-product from the wet process of palm oil milling which requires 5–7.5 tons of water to produce 1 ton of crude palm oil. Rich in fermentable sugars and nitrogenous compounds, palm oil mill effluent has been used as a fermentation media for microbial production of surfactin by *B. subtilis* ATCC 21332 [83]. Palm oil mill effluent has also been successfully used by *Nevskia ramose* NA3 for biosurfactant production [84]. Felled oil palm trunks contains glucose (approximately 85.2 g/L) in its sap, as well as low concentrations of sucrose, fructose, galactose, xylose and rhamnose, serving as a significant source for fermentation by yeast strains [85]. Pressed juice from oil palm fronds have also been reported to contain 53.95 g/L glucose and other sugars such as sucrose and fructose [86]. To utilize the oil palm trunk efficiently, it is first separated into sap and trunk fiber, where the sap is used directly while the trunk fiber is hydrolyzed to fermentable sugars using sulphuric acid [87]. The bioconversion of oil palm trunk waste into sugars via the lignocellulosic route indicates its promising potential as a renewable substrate for rhamnolipids production.

Several studies have been conducted to utilize olive mill waste as a carbon source in biosurfactant production [88]. Enzymatic hydrolysis of olive mill waste using a mixture of cellulases, hemicellulases and xylanase was carried out at 50 °C with agitation. Fermentation with *P. aeruginosa* and *B. subtilis* achieved 29.5 and 13.7 mg/L of rhamnolipids and surfactins, respectively [89]. Various *Pseudomonas* strains were found to be able to grow on olive oil mill effluent (OOME) mixed with 0.25 g/L sodium nitrate to produce 0.058 g rhamnolipids per g of OOME substrate [90]. It was demonstrated that *P. aeruginosa* could produce 5.1 g/L of rhamnolipids in a medium in which olive mill waste (25% v/v) was mixed with corn steep liquor (10% v/v) and sugarcane molasses (10% w/v) [91]. Another similar study reported that *P. aeruginosa* produced 191.46 mg/L rhamnolipids with 10% w/v olive mill waste while *B. subtilis* produced 3.12 mg/L surfactin with 2% w/v olive mill waste [92]. Coconut oil sludge and oil cake, another agro-industrial residue, has been used as a carbon source for rhamnolipids production by *P. aeruginosa* AMB AS7, achieving a maximum biosurfactant concentration of 5.53 g/L during 60 h of cultivation at 37 °C and 120 rpm [93].

#### Other agricultural residues

Agricultural residues such as barley bran, trimming vine shoots and corn cobs were used in biosurfactant production. To obtain the carbon source, the lignocellulosic residues was first treated with sulphuric acid and neutralizing with calcium carbonate. Yeast extract and corn steep liquor were served as nitrogen sources for *Lactobacillus pentosus* which could produce 0.71 and 0.28 g of biosurfactant per g of biomass from trimming vine shoots and barley bran husk hydrolysates, respectively [94]. In a similar study, trimming vine shoots were utilized by *Lactobacillus pentosus* hydrolysates for both lactic acid and biosurfactant production [95]. The trimming vine shoots first underwent acidic pre-hydrolysis to convert the hemicellulose polysaccharides (xylan, mannan and galactan) into corresponding monosaccharides (xylose, mannose and glucose). The hydrolyzed trimming vine shoots produced a fermentation medium containing 18 g/L xylose, 11.1 g/L glucose and 4.3 g/L of arabinose [95]. *Lactobacillus pentosus* could produce lactic acid and xylose to lactic acid (60%) and acetic acid (40%), and produces biosurfactants as part of the cell membrane [95]. Other bacteria such as *Acinetobacter*, *B. subtilis* and *Pseudomonas* are able to produce biosurfactants using such wastes [41]. It has been noted that all these mentioned carbon sources can be used for biosurfactant production by fungus which can produce different types of biosurfactant [96].

In addition to the aforementioned waste, Jackfruit, a tree that grows in tropical countries in Southeast Asia, comprises of 100–300 seeds in a single fruit. Jackfruit seeds have high carbohydrate and protein content, making up 10–15% of the total fruit mass, but are usually discarded. One study has utilized the jackfruit seeds as an abundant and low-cost substrate for biosurfactant production. *Deinococcus caeni* PO5 was shown to produce 3.12 g/L glycolipid biosurfactant when grown on an optimized medium containing jackfruit seed powder [97].

### Whey products

Dairy whey, milk serum which is generated after curdling of milk or coagulation of casein, is also a potential medium for rhamnolipids production. Whey contains lactose and proteins which can serve as carbon and nitrogen sources for microorganisms. *Pseudomonas aeruginosa* SR17 was able to produce rhamnolipids in the presence of paneer whey with a yield of 2.7 g/L [98]. The yield was further improved to 4.8 g/L when the paneer whey medium was supplemented with 2% glucose and mineral salts [98]. Another study showed that *P. aeruginosa* ATCC 10145 cultivated in raw cheese whey at 37 °C could produce 9.6 g/L rhamnolipids after 72 h [99]. *Lactobacillus pentosus* CECT-4023 was capable of producing biosurfactants in a medium containing cheese whey at 1.4 g/L [41, 100]. *Yarrowia lipolytica* MFW5, *Micrococcus luteus* MFW1 and *Burkholderia cepacia* MFW2 produced biosurfactants in whey wastewaters from the milk factory [101]. Whey tofu-a waste during tofu production was shown to be a viable medium for rhamnolipids production by *P. fluorescens* [102].

### Cassava

Cassava flour wastewater mixed with nutrient broth was served as a medium for rhamnolipids production by *P. fluorescens* [103]. When solid residues were removed from cassava wastewater and autoclaved for use as a growth medium for various *P. aeruginosa* strains, rhamnolipids yields between 169.9 and 300.3 mg/L were achieved [104]. One study showed an integrated process in which 10.5 g/L of biosurfactant was first produced by *Pseudozyma tsukubaensis*, and the resulting microbial cells were used to synthesize galactooligosaccharides from lactose [105].

## Challenges

Rhamnolipids are formed by hydrophilic l-rhamnose (mono-rhamnolipids) or l-rhamnopyranosyl-2-*O*-a-l-rhamnopyranoside (di-rhamnolipids) linked through an α-glycosidic bond to a hydrophobic moiety such as HAA, which are saturated or unsaturated fatty acids with alkyl chain length varying from 8 up to 16 carbon molecules. The biosynthesis and proportion of rhamnolipids types produced is dependent on nutritional and environmental conditions during microbial growth [106]. Challenges remain when sugars or sugar-containing wastes are used for rhamnolipids production.

### Rhamnolipids purification

The production of mono- or di-rhamnolipids depends on the regulation of the sequential induction of rhlAB and rhlC operons. When glucose is used as the carbon source, rhlAB and rhlC operons are simultaneously activated under the same QS mechanism. RhlA is involved in the production of HAAs, RhlB adds a rhamnose ring to the HAA precursor to form mono-rhamnolipids, and rhlC to catalyze the addition of the second rhamnose molecule to mono-rhamnolipids to synthesize di-rhamnolipids. However, in an oil-containing carbon source, rhlAB expression is not switched off, resulting in delayed transcriptional activation of rhlC to give rise to a major production of mono-rhamnolipids [107]. Purification of rhamnolipids can be achieved using conventional methods including solvent extraction or chromatography while these methods are time consuming. Foam fractionation is an efficient way for rhamnolipids purification [106, 108, 109] and the purity of rhamnolipids will be affected when oil-type carbon sources are used in fermentation as a carbon source as products have similar structures or form complexes with the substrates. Using sugar-type carbon sources will make the down-stream process relatively easy while it is still challenging to obtain pure mono- and di-rhamnolipid, respectively.

### Low yields from sugar-type substrates

Different metabolic pathways for hydrophilic and hydrophobic substrates synthesize different precursors for biosurfactant production. This provides an explanation for the various yields resulting from different carbon sources in the culture medium. When a glucose carbon source is used, both the lipogenic pathway and glycolytic pathway are suppressed by cell metabolism. Glucose is degraded until intermediate glucose-6-phosphate is formed. In order to form lipids, glucose has to be oxidized to pyruvate, then converted to acetyl-CoA, which is finally transformed to a fatty acid. However, when oils are used as the carbon source, gluconeogenesis is activated to produce sugars by oxidizing fatty acids through β-oxidation to acetyl-CoA. Polysaccharide precursor glucose-6-phosphate is then synthesized to form glucose. This gives rise to the hypothesis that oil-containing carbon sources produce higher yields of biosurfactants compared to sugars [110]. For example, *P. aeruginosa* and *P. fluorescens* were cultivated in mineral medium supplemented with vegetable oil, sucrose and octanoate and rhamnolipids production was the highest (174 mg/L) when vegetable oil was used as the carbon source [111]. The yield of rhamnolipid is higher when oil-type carbon sources are used in the culture media [112–114]. The yield of rhamnolipids reached 36.7 g/L when *P. aeruginosa* was grown in a medium containing sunflower oil [115]. It has been noted that altering the components of the medium can improve rhamnolipids yields when glucose was used as a carbon source [116].

### Fermentation condition optimization

Sugars derived from wastes contain several types of hydrocarbons, making fermentation control complicated. Studies comparing the variation within one type of carbon source were carried out. *Oceanobacillus* sp. BRI 10 was cultivated in a basal salt medium containing glucose, sugarcane juice, whey and commercial table sugar respectively, and sugarcane juice was found to produce the highest yield of biosurfactant [117]. High total sugar content (34–76% dry weight) was present in corn powder, potato peel powder and sugarcane bagasse compared to wheat straw, soybean powder and rice husk [118]. An alkaliphilic bacterium *Klebsiella* sp. Strain RJ-03 could produce 15.4 g/L biosurfactant in a medium supplied with corn powder [118]. Study has also shown that usage of a combination of sugar and oil sources enable optimal biosurfactant production. *Candida* (*Starmerella*) *bombicola* was cultivated on different lignocellulose hydrolysates—sweet sorghum bagasse and corn fiber, producing a sophorolipid yield of 3.6 and 1.0 g/L respectively. However, when soybean oil was added at 100 g/L, sophorolipid yield increased drastically to 84.6 and 15.6 g/L in sweet sorghum bagasse and corn fiber. This shows that cultures with both monomeric sugars and non-sugar compounds in biomass hydrolysates generated higher biosurfactant yield than a glucose medium with similar concentration [119]. A similar conclusion was drawn in another study, where the yeast *Candida bombicola* grown on low cost media based on sugarcane molasses and soybean oil produced 23.25 g/L of sophorolipids, comparable to conventional synthetic medium [120].

### Carbon sources affect rhamnolipids yields

It is known that different carbon sources give rise to various rhamnolipid yields [107]. Mixture of different types of carbon sources are used in rhamnolipid production. Mixtures of glucose and fatty acids with different chain lengths (C_12_–C_22_) and saturation were used to compare the effect of fatty acid substrates on rhamnolipids yield. Experimental results showed that 1% glucose and 0.25% stearic acid (C_18_) produced 2.1 g/L of rhamnolipids. The yields reached 14.3 g/L using 2% glucose and 2% stearic acid (C_18_). Generally, *P. aeruginosa* ATCC 9027 produced an increasing rhamnolipids yield with increasing fatty acid with chain length ranging from C_12_ to C_18_ [121]. When a sugar carbon source is selected, extensive experiments are required to obtain the optimal fermentation conditions.

### Sugar yield from lignocellulose

Despite intensive research to improve the release of fermentable sugars from lignocellulose, efficient hydrolysis of lignocellulose by enzymes remains challenging [122]. Lignin is adsorbed on cellulolytic enzymes, which blocks the access of cellulolytic enzymes to the substrate, hinders the removal of cellulase from cellulose, and reduces substrate hydrolysis [123]. Many studies have been carried out to improve the sugar yield from lignocellulose biomass. Addition of tea saponin to corncob residue during enzymatic hydrolysis enhanced the glucose yield from 34.29 to 46.28 g/100 g [124]. In another study, two-stage co-hydrolysis by *Trichoderma reesei* and *P. aeruginosa* BSZ-07 increased the production of reducing sugars (2.57 g/L) in rice straw by 15.2% [125]. Another example is elephant grass which requires pretreatment with sodium hydroxide and addition of Tween 80 surfactant to increase the efficiency of releasing reducing sugars for fermentation [126].

## Perspective

Rhamnolipids can be produced using sugars as carbon sources by bacteria such as *P. aeruginosa* and other non-pathogenic strains [45, 127–129]. It is known that less purification steps are required when sugars are used as carbon sources for rhamnolipids production. Although sugars such as glucose are commercially available as fermentation sources, these sugars are produced from food resources. When the produced rhamnolipids are used in large quantities, obtained sugars from a different resource is absolutely needed. To reduce production cost derived from carbon sources, sugar-containing wastes or a waste that can be converted into sugars are a good candidate as carbon sources for rhamnolipids production. The most abundant sugar containing material is cellulose [130, 131]. Therefore, wastes from agricultural products can be used as a carbon source for rhamnolipids production. The cellulose-containing wastes are normally much cheaper than sugar containing waste while converting cellulose to sugars is required before its application as carbon sources for rhamnolipids production. Converting cellulose to sugars has been well studied for decades [64, 66, 132]. Several methods can be used to convert cellulose into fermentable sugars [133–136]. Therefore, it is feasible to use these wastes for rhamnolipids production. These wastes are very promising when the rhamnolipids product is used in some fields which does not require purified biosurfactant.

In addition to rhamnolipids, other biosurfactants can be produced using cellulose-containing wastes as carbon sources [137, 138]. The accumulated experience is also useful for guiding rhamnolipids production using strains such as *P. aeruginosa*. Other parameters that can be used to reduce rhamnolipids production cost include using more powerful strains, an optimized fermentation procedure, an efficient sugar processing strategy, and an easy purification step [15]. Nonetheless, using sugars derived from cellulose-containing material as a carbon source is a good choice for rhamnolipids production at a low cost. More work needs to be carried out to explore a suitable conditions for large-scale rhamnolipids production using sugar-containing wastes, especially for the wastes that requires pretreatment.
